# Estimating the Prevalence of Childhood Obesity in Alaska Using Partial, Nonrandom Measurement Data

**DOI:** 10.5888/pcd13.150429

**Published:** 2016-03-24

**Authors:** Erik Everson, Myde Boles, Karol Fink, Rebecca Topol, Andrea Fenaughty

**Affiliations:** Author Affiliations: Myde Boles, Program Design and Evaluation Services, Multnomah County Health Department and Oregon Public Health Division, Portland, Oregon; Karol Fink, Rebecca Topol, Andrea Fenaughty, Alaska Section of Chronic Disease Prevention and Health Promotion, Anchorage, Alaska.

## Abstract

Although monitoring childhood obesity prevalence is critical for state public health programs to assess trends and the effectiveness of interventions, few states have comprehensive body mass index measurement systems in place. In some states, however, assorted school districts collect measurements on student height and weight as part of annual health screenings. To estimate childhood obesity prevalence in Alaska, we created a logistic regression model using such annual measurements along with public data on demographics and socioeconomic status. Our mixed-effects model-generated prevalence estimates validated well against weighted estimates, with 95% confidence intervals overlapping between methodologies among 7 of 8 participating school districts. Our methodology accounts for variation in school-level and student-level demographic factors across the state, and the approach we describe can be applied by other states that have existing nonrandom student measurement data to estimate childhood obesity prevalence.

## Background

In 2011–2012, approximately 1 in 6 (17.7%) US children aged 6 to 11 were obese (1). Compared with their nonobese peers, these children are more likely to experience low self-esteem and depression (2), discrimination by other children (3), and academic struggles (4); they are also more likely to become obese adults (5,6). The ability to monitor childhood obesity prevalence at the state and local levels is critical for state public health programs to assess trends and the success or failure of interventions.

In estimating obesity prevalence among children, the limited availability or reliability of data on height and weight often presents a challenge. State data on children’s self-reported or parent-reported height and weight are available from national surveys (7,8), but these data are unreliable and often unavailable for younger ages (9,10). Among a sample of children aged 6 to 11, one study (11) found that obesity prevalence estimated from parent-reported height and weight was as much as double the actual measured prevalence.

Direct measurement of children’s height and weight is ideal for estimating rates of childhood obesity, but collecting a comprehensive or random sample involves many barriers. Public school students offer a convenient proxy for school-aged children, but measuring the height and weight of students may still require staff time, travel, and training expenses, along with additional staff time for coordination between school districts and the state. Only about a dozen US states have implemented comprehensive systems for screening body mass index (BMI) in selected grades (12). Among national surveillance efforts, only the National Health and Nutrition Examination Survey (NHANES) measures the height and weight of children directly. NHANES is designed to produce national estimates for the US population and some subgroups, however, and is not suitable by itself or of sufficient sample size for obtaining state or local estimates (13).

One study (14) recently used NHANES data to build a demographics-based obesity model and then applied local demographic data from other sources to estimate childhood obesity rates in Georgia. Although this method could be applied in many areas of the United States, it cannot be used in states that have unusual geography or whose populations have unique demographic characteristics that are not reflected in NHANES data. In Alaska, American Indian or Alaska Native (AIAN) children are the majority in half (51.9%) of the state’s school districts (15), but NHANES data sets are not large enough to provide estimates for this relatively small and geographically specific population and do not offer an AIAN race/ethnicity category (16). Furthermore, even if national AIAN data were available, the data might not accurately represent Alaska’s predominately Alaska Native AIAN population. This problem is not unique to Alaska; other regions of the United States have large populations of racial/ethnic subgroups that are combined into nonspecific, nonrepresentative racial/ethnic categories; for example, the weight status of Pacific Islanders or Cuban Americans may not be accurately represented by the NHANES categories of “non-Hispanic Asian” or “other Hispanic” (17,18).

Modeling obesity prevalence on national data is not suitable for Alaska because of the state’s unique population of Alaska Native people and remote geography. Like most states, Alaska also lacks a comprehensive measurement system in schools from which to directly estimate childhood obesity prevalence. Several Alaska school districts do, however, collect height and weight measurements annually for selected grades. To estimate childhood obesity prevalence in Alaska, we created a logistic regression model that uses these measurements and accounts for variation in school-level and student-level demographic factors across the state. The objective of this article was to describe our methodology, which can be used by other states that have existing, partial, nonrandom data on student height and weight measurements to estimate obesity prevalence.

## Methods

### Collecting data on height and weight measurements and demographics

**Student data.** Our modeling data set combined student height and weight measurements collected by 8 Alaska school districts during the 2013–2014 school year. Measurements were conducted by school nurses and public health nurses as part of routine health screenings and an ongoing state program to monitor student overweight and obesity in each district. Although we received some measurements for all grades (pre-kindergarten through grade 12), we limited our analysis to only those grades measured in all districts: kindergarten, grade 1, grade 3, grade 5, and grade 7 (hereinafter called “K–7”). Although the 8 participating school districts represent only 15% of Alaska’s 54 school districts, they include the state’s largest enrollment districts and together have 62.8% of the state’s K–7 students.

School district staff members provided height and weight measurements for 83.9% (n = 26,576) of enrolled K–7 students in the 8 districts. Measurement data also included data on race/ethnicity (white, black, Asian, Pacific Islander or Native Hawaiian, American Indian, Alaska Native, multirace, and Hispanic), sex, grade, and age in months at date of measurement for each student. We excluded incomplete records and those with biologically implausible values for height, weight, or BMI, using criteria by sex and age developed by the Centers for Disease Control and Prevention (CDC) (19). After all exclusions, our net measurement rate for K–7 students in the 8 participating districts was 82.8% (n = 26,206) (Table 1). 

**Table 1:** Sample Observations, Students in Kindergarten, Grade 1, Grade 3, Grade 5, and Grade 7, by Selected Characteristics, Alaska, School Year 2013–2014

Category	No. of K–7 Students Enrolled	No. of Valid Measurements	Measurement Rate, %^a^	% of Sample^b^	% of Alaska’s K–7 Enrollment
**District**					
Anchorage	18,885	16,111	85.3	61.5	37.5
Matanuska–Susitna Borough	6,645	4,996	75.2	19.1	13.2
Kenai Peninsula Borough	3,344	3,027	90.5	11.6	6.6
Kodiak Island Borough	926	678	73.2	2.6	1.8
Ketchikan Gateway Borough	818	729	89.1	2.8	1.6
North Slope Borough	719	440	61.2	1.7	1.4
Alaska Gateway	171	75	43.9	0.3	0.3
Petersburg City	155	150	96.8	0.6	0.3
All sample districts	31,663	26,206	82.8	100.0	62.8
**Sex**					
Male	15,207	13,499	82.0	51.5	51.8^c^
Female	16,456	12,707	83.6	48.5	48.2^c^
**Race/ethnicity**					
Non-Hispanic white	16,473	13,546	82.2	51.7	48.9^c^
Non-Hispanic American Indian/Alaska Native	4,304	3,416	79.4	13.0	23.7^c^
Other	10,886	9,244	84.9	35.3	27.4^c^
**Grade**					
Kindergarten	6,654	6,048	91.7	23.1	21.1^c^
Grade 1	6,599	4,753	76.0	18.1	20.9^c^
Grade 3	6,256	5,282	88.2	20.2	19.7^c^
Grade 5	5,992	4,942	80.2	18.9	19.0^c^
Grade 7	6,162	5,181	77.9	19.8	19.3^c^

a The number of students for whom height and weight measurements were taken divided by the number of students enrolled.

b Percentages for each category may not total 100 because of rounding.

c These values represent the percentage distribution in Alaska’s K–7 enrollment; percentages for each category may not total 100 because of rounding.

**Socioeconomic data.** As a proxy for school-level socioeconomic status (SES), we obtained data on the percentage of students at each school eligible for the National School Lunch Program (NSLP) (20). Students eligible for this program had family incomes at or below 185% of federal poverty guidelines. Our SES data also indicated which schools did not participate in NSLP; the prevalence of obesity at these nonparticipating schools was lower than the prevalence at participating schools that had low percentages of eligible families (ie, schools with high SES).

**Additional derived variables.** Our modeling data set contained several derived variables in addition to the student height and weight measurement and demographic data provided by schools. We computed BMI percentile using a CDC-developed SAS program (21) and created a binary obesity variable indicating students at or above the 95th percentile for age and sex (ie, CDC’s definition of obesity). We recoded student data on race/ethnicity into 3 categories to ensure adequate sample size for each: non-Hispanic white, non-Hispanic AIAN, and other. The racial composition of the “other” category varied significantly by school district.

We also created several school-level variables. Because of obesity’s associations with poverty and environmental factors (sociocultural and physical), the racial/ethnic composition of a community can be associated with childhood obesity because of factors other than the race/ethnicity of the children (22). To account for community race/ethnicity, we created 2 variables indicating the percentage of enrolled AIAN students and the percentage of enrolled other-race students at each school. Using our NSLP eligibility data, we created 3 school-level SES categories: low SES (≥45% eligibility), high SES (<45% eligibility), and nonparticipating school. We chose 45% as a cutoff value because it corresponds with Alaska’s eligibility criteria for low-income school funding. Finally, we also created variables ranking school size and indicating metropolitan area or nonmetropolitan area, although neither variable was included in our final model.

### Modeling student obesity

**Model specification.** We created a mixed-effects logistic regression model to predict the prevalence of student obesity based on demographic factors while accounting for data clustering and random effects at the school level. We developed all aspects of the model using Stata 13.1 software (StataCorp LP), using the “meqrlogit” command with default options. 

Our model’s dependent variable was student obesity status. Independent variables included grade 1, grade 3, grade 5, grade 7, male sex, AIAN race, and “other” race (all student-level), along with the percentage of school population that was AIAN, the percentage of the school population that was “other” race, high-SES school, and low-SES school. To account for multicollinearity, we centered each student-level race variable as follows: percentage of school population AIAN (centered) *= *AIAN race (0/1) − percentage of school population AIAN. For example, for an Alaska Native student in a school whose student population was 30% AIAN, the centered student-level AIAN race variable would be calculated as the following: 1 − 0.3 = 0.7. Finally, our model included a random-effects term to describe school-level variation in obesity beyond what was accounted for by the other independent variables.

**Model application.** To apply our model toward estimating statewide obesity rates, we created a second data set representing all Alaska public schools. This data set included official school-level enrollment counts for the 2013–2014 school year by grade, sex, and race/ethnicity provided by the Alaska Department of Education & Early Development, as well as school-level SES and the same set of derived variables used in our model. After building our model, we estimated statewide K–7 obesity prevalence by applying it to this data set.

**Model validation.** We used a jackknife validation procedure: for each participating school district, we re-ran our model without that district’s measurement data and then applied the district’s enrollment data to estimate its obesity prevalence. We also created alternative estimates by computing weights to reflect each district’s enrollment by grade, sex, and race/ethnicity and then applying these weights to each district’s measured obesity prevalence. To validate our model, we compared the model-generated estimates to the weighted estimates. Because of the large size and uniquely-metropolitan nature of Anchorage School District in our sample data, we randomly divided Anchorage schools into approximate halves and treated each as a separate district for validation purposes. To describe the portion of total variance due to variance among schools, we calculated the variance partitioning coefficients for each model as level-2 variance divided by the sum of level-2 variance and π^2^/3 (23).

## Results

**Statewide obesity estimate.** By building a regression model using available measurement and demographic data and applying it to statewide enrollment data, we estimated that 19.4% of Alaska students in kindergarten, grade 1, grade 3, grade 5, and grade 7 were obese in the 2013–2014 school year. All of the model’s independent variables, except high-SES school, were significant at *P* ≤ .05 level (Table 2).

**Table 2:** Weight-Estimated Obesity Prevalence and Model-Estimated Obesity Prevalence, by Selected Characteristics, Students in Kindergarten, Grade 1, Grade 3, Grade 5, and Grade 7, School Year 2013–2014

Category/Group	% (95% CI)		
	Statewide (All Districts), Modeled Estimate^a^	8 Measured Districts Only	
		Modeled Estimate^a^	Weighted Estimate^b^
**Alaska state total**	19.4 (19.0–19.8)	17.1 (16.7–17.5)	17.2 (16.1–18.3)
**Sex**			
Male	20.9 (20.2–21.5)	18.5 (17.8–19.1)	18.6 (17.4–19.8)
Female	17.8 (17.2–18.4)	15.7 (15.0–16.3)	15.7 (14.5–17.0)
**Race/ethnicity**			
Non-Hispanic white	11.9 (11.4–12.5)	12.1 (11.5–12.6)	12.3 (11.4–13.2)
Non-Hispanic American Indian/Alaska Native	32.0 (30.6–33.5)	24.1 (22.7–25.5)	24.0 (22.0–26.1)
Other	21.8 (20.4–23.1)	22.0 (20.6–23.4)	22.0 (20.6–23.5)
**Grade**			
Kindergarten	15.2 (14.0–16.3)	13.1 (12.0–14.3)	13.3 (12.0–14.6)
Grade 1	16.7 (15.7–17.6)	14.5 (13.5–15.4)	14.7 (13.1–16.4)
Grade 3	20.0 (18.9–21.0)	17.6 (16.6–18.6)	17.5 (16.0–19.1)
Grade 5	21.1 (21.0–23.2)	19.9 (18.8–21.0)	20.0 (18.4–21.6)
Grade 7	23.7 (22.6–24.8)	21.0 (19.9–22.1)	21.2 (19.0–23.5)

Abbreviation: CI, confidence interval.

a Logistic regression model predicting the prevalence of student obesity based on independent variables representing student-level sex, race/ethnicity, and grade, and school-level race/ethnicity distribution, socioeconomic status, and random effects.

b Measured obesity prevalence weighted up to district enrollment totals by sex, race/ethnicity, and grade.

**Validation results.** Confidence intervals for weighted estimates overlapped with those for modeled estimates for all school districts except Kenai Peninsula (Table 3). The mean square error between methodologies was 9.2%. The jackknife model estimates summed to a prevalence estimate of 17.1% for all measured districts, compared with 17.2% for the weighted estimates. The estimates by sex, race/ethnicity, and grade for the 8 measured districts in our primary model were all within 0.2% of their weighted estimates (Table 2). 

**Table 3:** Model-Estimated Obesity Prevalence and Weight-Estimated Obesity Prevalence, Students in Kindergarten, Grade 1, Grade 3, Grade 5, and Grade 7, by District, Alaska, School Year 2013–2014

School District	Weighted Estimate, % (95% CI)^a^	Jackknife Models^b^	
		Modeled Estimate % (95% CI)	School-Level Variance Partitioning Coefficient^c^
Anchorage part A	18.6 (16.4–21.0)	17.9 (17.0–18.7)	0.0153
Anchorage part B	17.1 (15.0–19.3)	17.6 (16.8–18.4)	0.0179
Matanuska–Susitna Borough	14.0 (12.3–15.8)	14.3 (13.3–15.3)	0.0151
Kenai Peninsula Borough	16.5 (14.7–18.5)	12.4 (11.1–13.7)	0.0125
Kodiak Island Borough	15.0 (9.5–22.9)	21.1 (18.4–23.8)	0.0129
Ketchikan Gateway Borough	21.4 (17.7–25.5)	19.9 (16.9–22.9)	0.0129
North Slope Borough	31.8 (19.1–47.9)	35.2 (30.9–39.5)	0.0130
Alaska Gateway	25.8 (22.4–29.6)	29.6 (19.8–39.5)	0.0145
Petersburg City	17.5 (16.4–18.7)	17.5 (11.6–23.5)	0.0146

Abbreviation: CI, confidence interval.

a Measured obesity prevalence weighted up to district enrollment totals by sex, race/ethnicity, and grade.

b Logistic regression models predicting the prevalence of student obesity based only on data from school districts other than the 8 districts participating in this study and variables representing student-level sex, race/ethnicity, and grade and school-level race/ethnicity distribution, socioeconomic status, and random effects.

c Variance partitioning coefficients for each model were computed by dividing level-2 variance by the sum of level-2 variance and π^2^/3 (23).

## Discussion

We created a logistic regression model to estimate the prevalence of obesity among Alaska students based on a partial, nonrandom set of height and weight measurements and data on student-level and school-level demographic factors. Our method is straightforward and can be easily implemented by other jurisdictions with incomplete school measurement data. This method is also capable of describing unique local populations that are not adequately represented in national surveys such as NHANES. To our knowledge, our study is the first to estimate statewide obesity prevalence among children using locally collected, nonrandom measurement data.

Our results show a large disparity in statewide obesity prevalence between non-Hispanic white students (11.9%) and racial/ethnic minority students, particularly AIAN students (32.0%), in grades K–7. This disparity is consistent with our model coefficients and with previous findings across multiple years (24) in which the prevalence of obesity among AIAN students consistently exceeded that among white students. The burden of obesity among AIAN students is typically largest in rural areas, where AIAN students are more concentrated.

Our model indicates that obesity among AIAN students is significantly higher statewide (32.0%) than in the 8 districts for which we had measurements (24.1%); these 8 tend to be more metropolitan than other districts in the state. This difference highlights the value of our modeling approach, in that the prevalence of statewide obesity would be underestimated by either 1) weighting up (disproportionately metropolitan) measured obesity prevalence to state enrollment totals or 2) using a model such as ours but without AIAN-specific measurements.

We developed our model to account for Alaska’s unique population. Outside of the Anchorage/Matanuska–Susitna area, 37% of K–7 students are AIAN, 31% of schools have fewer than 50 students, and many Alaska communities are inaccessible by road. Accordingly, our initial model included several independent variables to designate location-related differences. School-level variables for percentage of school population that was AIAN and percentage of school population that was “other” race were significant in our model (*P* < .001) and predicted obesity beyond student-level demographic factors including race/ethnicity. Independent variables for school size and metropolitan/nonmetropolitan classification (not used in our final model) were not significant (at *P* ≤ .05) when used along with terms indicating school-level race/ethnicity composition. In our sample, then, racial/ethnic composition better captured the variation among schools in how student demographics translated to obesity than did factors such as school size or metropolitan/nonmetropolitan classification.

Although we arrived at a particular set of independent variables in modeling Alaska student obesity prevalence, other states undoubtedly have their own unique sets of factors to best describe how demographics associate with obesity. States using a similar approach may wish to account for their own distinct racial/ethnic populations, regions, urban or rural classifications, public or private schools, or any other student-level, school-level, or area-level variable for which reliable data are available.

The method we described can also be applied to produce prevalence estimates for various metrics (eg, overweight, severe obesity) or various areas (eg, region, school district, school). As with state-level estimates, accurate results depend on a measurement sample that is of sufficient size and is representative of the variation in the population being estimated. As with AIAN students in our model, subpopulations can be underrepresented in the measurement sample (relative to the population being estimated), provided they are identified in the model.

Our study has several limitations. Although we developed our model with data from 8 districts and 183 schools, most (61.5%) of our model development sample was from the state’s largest district, Anchorage School District. Our modeling approach accounted for this by distinguishing among schools of varying racial minority distributions and SES levels, and schools representing a broad range of each factor were included in our model development sample. Nonetheless, Anchorage has only 37.5% of Alaska’s K–7 enrollment, and a sample more equally distributed throughout the state would have been preferable.

Our measurement data included 8 race/ethnicity classifications, but we collapsed 5 of these categories (black, Hispanic, Asian, Native Hawaiian/Pacific Islander, and multirace) into “other” because of small sample sizes. The students in these categories accounted for 34% of K–7 enrollees in districts that provided measurements but only 16% in districts that did not. The racial/ethnic composition of “other” students varied significantly by district, but the overall composition was similar between the set of districts that provided measurements and the set that did not. Nonetheless, additional race/ethnicity categories would have been ideal had we had ample sample sizes, especially in producing estimates for individual districts.

Finally, the height and weight measurements we used were not collected through a statistically valid sampling procedure or with regularly calibrated equipment but rather as part of schools’ routine health screening process. Nonetheless, participating schools tried to measure all K–7 students, so we have no reason to believe the sample of students was biased toward or against obese students (25). Our method does not account for potential weight bias caused by children dropping out of school, a concern that would increase in studies of older students.

Although a random or comprehensive sample is ideal for estimating the prevalence of childhood obesity, there are many barriers to collecting such a sample, including lack of funding, lack of on-site school nurses, and challenges in coordination between school districts and the state. Our method offers a straightforward way for states with existing, partial, nonrandom school measurement data to estimate childhood overweight or obesity prevalence. With sufficient locally representative measurement data, our method is capable of producing accurate estimates of obesity prevalence for children in elementary school and middle school, and can be adapted to account for unique populations and regional variations in how demographic factors relate to weight status.
